# Supplementary material for: Seroprevalence, incidence estimates, and environmental risk factors for dengue, chikungunya, and Zika infection amongst children living in informal urban settlements in Indonesia and Fiji
Source: BMC Infect Dis. 2025 Jan 13;25:51. doi: 10.1186/s12879-024-10315-1 (PMC11727629; doi:10.1186/s12879-024-10315-1)
Supplement: Supplementary file 1 — Supplementary Material 1. [file 12879_2024_10315_MOESM1_ESM.docx]

**Supplementary Material:**

| Supplementary Table. Survey questions | |
| --- | --- |
| *Survey question* | *Survey answer choices* |
| In the last 3 months, has the child ever consumed breast milk? | Yes No |
| Is the child currently being breastfed? | Yes No |
| What is your main ethnicity? | *Fiji:* I Taukei Indo Fijian Other  *Indonesia:* Makassar Bugis Toraja Mandar Luwu Ende Maumere Atoni/Dawan Manggarai Sumba Javanese Bali Chinese Mixed Other Other |
| Is everyone in your household [ethnicity of respondent]? | Yes No *If response was no, household was labeled as mixed ethnicity. |
| What is your main religion? | *Fiji:*  Christian Lotu Vakarisito Hindhu Islam Other No religion  Indonesia: Islam Catholic Protestant Hindu Buddha Confucian Other religion No religion |
| Is everyone in your household [religion of respondent]? | Yes No *If response was no, household was labeled as mixed religion. |
| In the past 6 weeks, how often have you encountered mosquitoes trying to bite you inside your house? | *Coded as "many"* Very often, every day Often, several times a week  *Coded as "not many"* Occasionally, one a week or less Never |
| Is your garbage routinely collected and taken away from your neighborhood? | *Coded as "garbage collected"* Yes, always  *Defer to next question* Yes, sometimes No, never |
| What other ways do you dispose of your garbage? (If garbage collection was sometimes or never in previous question) | *Coded as "garbage collected"* Neighborhood collection point  *Coded as "garbage not collected"* Dumped in the yard/garden/vacant land within the settlement Dumped in the yard/garden/vacant land outside the settlement Dumped in a waterway/drain within the settlement  Dumped in a waterway/drain outside the settlement |
| Does anyone in your house grow plants? This can include plants grown in your house, in a garden, or in croplands within the settlement. | Yes No |
| Over the last 3 months has the area directly outside or under your house flooded? | Yes No **Answering yes to either of the flood question was coded as "experienced flooding"* |
| Over the last 3 months, did your house flood, with water entering the house? | Yes No **Answering yes to either of the flood question was coded as "experienced flooding"* |
| What is the main flooring type used in this house (select all that apply)? | *Coded as "porous" if included any of the following materials:* Wood/boards Soil / dirt  *Coded as "non-porous" if only contained the following materials:* Ceramic / tiles / terrazzo Laminate (plastic) / linoleum Concrete Granite / stone Gravel Bricks |
| What is the main material used in the outer wall of this house (select all that apply)? | *Coded as "porous" if included any of the following materials:* Wood or plywood Bamboo, woven or mat Tent or tarpaulin  *Coded as "non-porous" in only contained the following materials:* Masonry (cement/prefabricated bricks/concrete blocks) Tin or corrugated iron Ceramic tiles |
| Stores water | Do you ever store water from XX water source, for example, in a container or a jug, in your house? **If answered yes to this question for any water source used by the household, coded as "stores water"* |
